# Supplementary figures and images for: Understanding and Exploiting Post-Translational Modifications for Plant Disease Resistance
Source: Biomolecules. 2021 Jul 30;11(8):1122. doi: 10.3390/biom11081122 (PMC8392720; doi:10.3390/biom11081122)

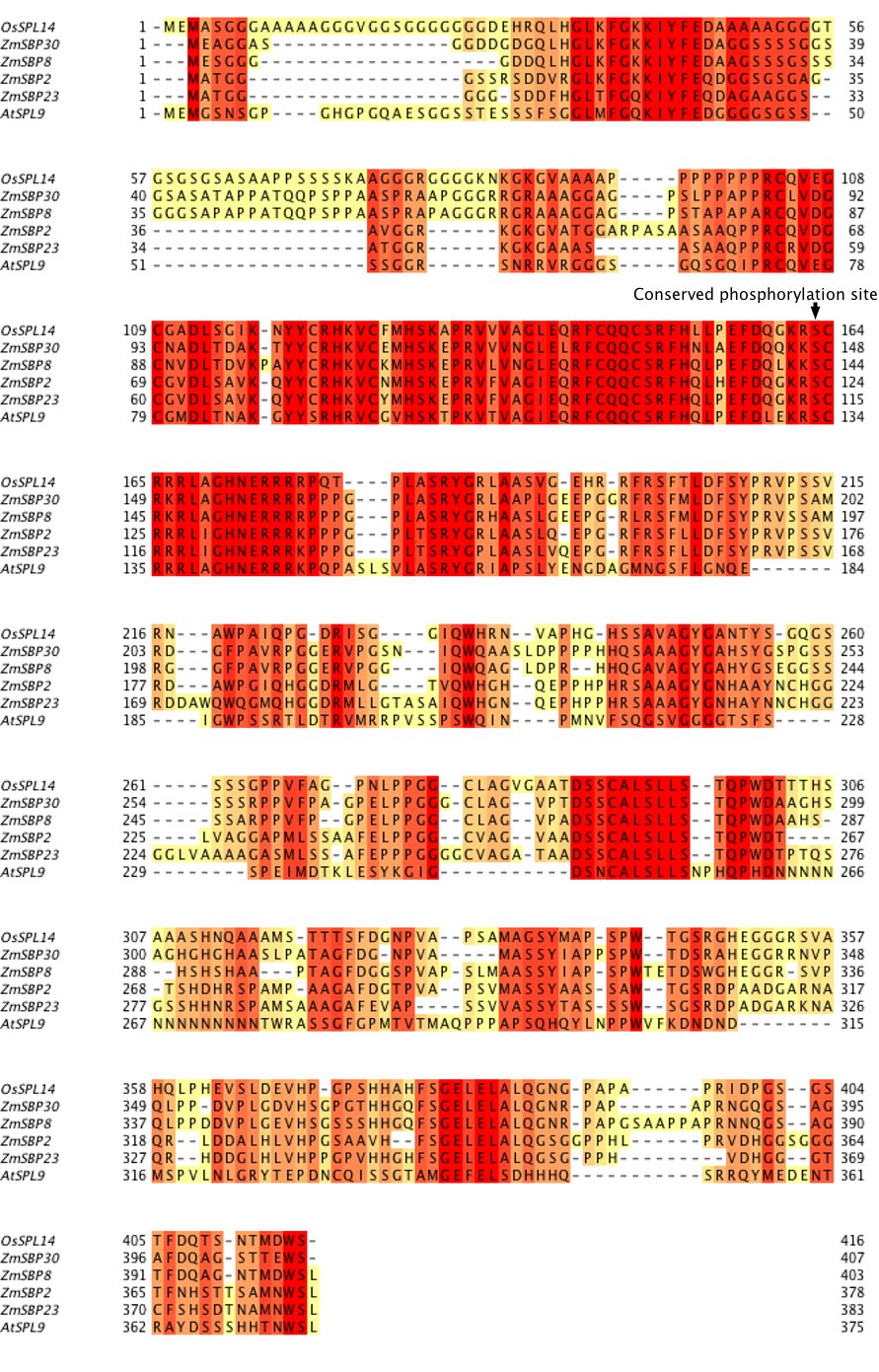

Supplement: Supplementary file 1 [file biomolecules-11-01122-s001.zip › biomolecules-1309773-supplementary.jpg]
